# Consequence of Paradigm Shift with Repeat Landscapes in Reptiles: Powerful Facilitators of Chromosomal Rearrangements for Diversity and Evolution (Running Title: Genomic Impact of Repeats on Chromosomal Dynamics in Reptiles)

**DOI:** 10.3390/genes11070827

**Published:** 2020-07-21

**Authors:** Syed Farhan Ahmad, Worapong Singchat, Maryam Jehangir, Thitipong Panthum, Kornsorn Srikulnath

**Affiliations:** 1 Laboratory of Animal Cytogenetics and Comparative Genomics (ACCG), Department of Genetics, Faculty of Science, Kasetsart University, 50 Ngamwongwan, Chatuchak, Bangkok 10900, Thailand; farhan.phd.unesp@gmail.com (S.F.A.); worapong.si@ku.th (W.S.); maryam.bioinfo.unesp@gmail.com (M.J.); thitipong.pa@ku.th (T.P.); 2 Special Research Unit for Wildlife Genomics (SRUWG), Department of Forest Biology, Faculty of Forestry, Kasetsart University, 50 Ngamwongwan, Chatuchak, Bangkok 10900, Thailand; 3 Integrative Genomics Lab-LGI, Department of Structural and Functional Biology, Institute of Bioscience at Botucatu, São Paulo State University (UNESP), Botucatu 18618-689, Brazil; 4 Center for Advanced Studies in Tropical Natural Resources, National Research University-Kasetsart University, Kasetsart University, Bangkok 10900, Thailand; 5 Center of Excellence on Agricultural Biotechnology (AG-BIO/PERDO-CHE), Bangkok 10900, Thailand; 6 Omics Center for Agriculture, Bioresources, Food and Health, Kasetsart University (OmiKU), Bangkok 10900, Thailand; 7 Amphibian Research Center, Hiroshima University, 1-3-1, Kagamiyama, Higashihiroshima 739-8526, Japan

**Keywords:** chromosome, genome, karyotype, sex chromosome, amniote

## Abstract

Reptiles are notable for the extensive genomic diversity and species richness among amniote classes, but there is nevertheless a need for detailed genome-scale studies. Although the monophyletic amniotes have recently been a focus of attention through an increasing number of genome sequencing projects, the abundant repetitive portion of the genome, termed the “repeatome”, remains poorly understood across different lineages. Consisting predominantly of transposable elements or mobile and satellite sequences, these repeat elements are considered crucial in causing chromosomal rearrangements that lead to genomic diversity and evolution. Here, we propose major repeat landscapes in representative reptilian species, highlighting their evolutionary dynamics and role in mediating chromosomal rearrangements. Distinct karyotype variability, which is typically a conspicuous feature of reptile genomes, is discussed, with a particular focus on rearrangements correlated with evolutionary reorganization of micro- and macrochromosomes and sex chromosomes. The exceptional karyotype variation and extreme genomic diversity of reptiles are used to test several hypotheses concerning genomic structure, function, and evolution.

## 1. Introduction

Over 150 years ago, Darwin and Wallace first proposed the theory of natural selection requiring variation among species individuals and stable inheritance from generation to generation [1,2]. However, the mechanism of this variation remained unclear until Dobzhansky observed the occurrence of chromosomal changes among species in a *Drosophila* lineage [3]. It is these rearrangements that supplement the raw materials for evolution, thereby enabling populations to evolve rapidly under natural selection [4]. Evolutionary mechanisms include variation in chromosome size, composition, and number between and within species, which has been termed “stasipatric speciation” [5]. Such chromosomal variation is also associated with reproductive isolation and outbreeding depression [6,7,8,9]. Approximately 320 million years ago, amniotes diverged into two major lineages comprising Synapsida, including all living mammals, and Sauropsida, including all extant non-avian reptilian and avian species [10,11,12,13]. Extensive diversity in chromosomal changes is observed among different lineages of non-avian reptiles, and a chromosomal evolutionary model is required to elucidate the source, timing, and types of changes between species. Sauropsids include Archosauromorpha (birds, crocodiles, and turtles) and Lepidosauromorpha (tuataras and squamate reptiles). Higher chromosome variability is observed among squamate reptiles, which show substantial variation in chromosome numbers (2*n* = 30–50). Their karyotypes can be categorized into two groups, consisting of those with few or no dot-shaped microchromosomes with an undetectable centromere, as found in Lacertidae and Gekkota, and those with macrochromosomes and many microchromosomes, as commonly observed in Scincoidea (skinks) and Episquamata (iguania, snakes, and monitor lizards) excluding Lacertidae [14,15,16,17,18,19,20,21]. A karyotype comprising a small number of macrochromosomes and many microchromosomes is also observed in birds and turtles [14,22,23,24,25,26]. By contrast, the karyotype of crocodiles is composed of chromosomes with a small number of large chromosomes and the absence of dot-shaped microchromosomes [8,27,28,29,30,31].

Chromosomal rearrangements often occur in combinations of different types as the source of karyotypic variation, and advances in omics technology enable elucidation of fine-scale changes in chromosome structure. Research interest has shifted from gross chromosomal rearrangements to smaller cryptic changes, such as segmental duplication and insertions/deletions [32,33]. These rearrangements can reshuffle genes, termed the ‘position effect’, through the location of regulatory elements and deletion of several genes or a portion of a single gene. Recent genomic sequencing projects involving several reptiles have led to an improved understanding of the substantial difference in proportions of genomic elements between functional genes and repeats [33,34,35,36]. Surprisingly, a similar number of genes is observed across amniotes, whereas different proportions and types of repeats are observed [37]. One emerging hypothesis is that a variety of chromosomal rearrangements are mediated through the transposition of interspersed repeats, such as transposable elements (TEs), and expansion of tandemly organized satellite sequences, which act as catalysts to drive genome evolution [20,21,26,29,36,38,39,40,41,42,43,44,45,46]. The correlation between repeats and chromosomal rearrangements must be investigated in the context of the diversity and evolution of reptilian lineages. To date, genome-wide characterization of repeats (‘repeatomics’) has focused on certain animal groups, such as mammals [47,48], with scant attention given to reptilian genomes [49]. Following completion of the first two mammalian genome sequencing projects involving mouse and humans [50,51], a decade passed before publication of the first reptilian genome, that of the green anole (*Anolis carolinensis*) in 2011 [52]. In this modern era of next generation sequencing (NGS), the number of sequenced mammalian genomes is considerably greater than that of reptiles, although the total number of reptilian species is almost four times higher than that of mammalian species [53]. However, genomic assemblies for several reptiles are in progress, which will provide novel resources for high-throughput repeatomic analyses of diverse lineages [49]. As of May 2020, the National Center of Biotechnology Information genome database included 64 publicly available assembled genomes (https://www.ncbi.nlm.nih.gov/genome/?term=reptiles), and this number is expected to rise rapidly. This advance has heralded renewed interest in several questions that link reptilian genomes and repeats, including (i) how does the proportion of repeats in the reptilian genome differ from that of other amniotes, (ii) in which specific genomic repeats do reptiles differ from other amniotes, (iii) how do genomic repeat contents differ among reptilian lineages, (iv) what potential mechanisms affect karyotypic evolution in reptiles through genomic repeats, and (v) is repeatomic diversity correlated with the extensive chromosomal variation seen in reptiles. Here, we review evidence pertaining to different repeat profiles in reptiles obtained from molecular cytogenetics and comparative genomics research. We highlight data for key species and present a comparative overview of repeat landscapes in different reptilian lineages. The dynamics of repeat-mediated rearrangements and their evolutionary impact on reptilian genome reorganization are discussed.

## 2. Diversity of Repeats in Reptiles Versus Other Amniotes

**Figure 1:**
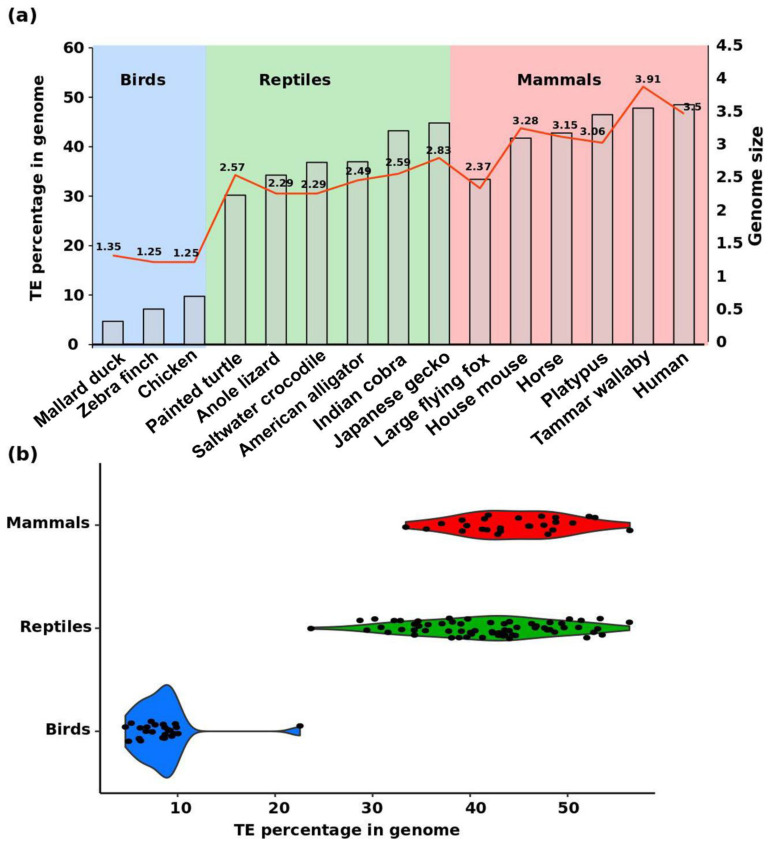
Percentage of transposable elements (TEs) in the genome representative amniotes. The bird genome contains the lowest percentage of TEs and genome size compared with the genomes of mammals and reptiles. (**a**) Bar chart shows the TE percentage of different birds (blue), reptiles (green) and mammals (red) and the red line indicates the genome size. (**b**) Distribution of total TE percentage in the genome of different species across reptiles, mammals and birds. Each dot represents a species. The species list is given as Supplementary Dataset S1.

Amniotes exhibit substantial variation in genomic composition, structure, and size; however, the number of protein-coding genes is similar across diverse amniote lineages [37]. Important features that contribute to such genomic variation are the diversity and different proportions of TEs [37,54,55]. Transposable elements, collectively termed the ‘mobilome’, constitute the major portion of the genome and are capable of self-replication and/or multiplication [56]. Transposable elements play an important role in genome evolution and contribute to a variety of genetic novelties, such as gene regulation for reshaping phenotypic diversity in the lineage [57,58]. Long terminal repeats (LTRs) and SINE-VNTR-Alu retrotransposons affect functional gene expression in primates and drive evolutionary divergence [58]. By contrast, TEs can have certain detrimental effects on the host genome as a result of direct insertions in functional genes or indirectly through non-homologous recombination [59,60]. Although amniotes contain the majority of known eukaryotic TEs, substantial variation in copy number, nucleotide sequence, and evolutionary age have been identified among lineages [55,61]. Transposable elements constitute a higher proportion of mammalian, squamate reptile, and turtle genomes compared with that of avian genomes (Figure 1). Whole-genome repeat annotations in birds and mammals indicate 1.7- to 2.2-fold variation in number of TEs among species [37]. Mammalian genomes differ substantially in TE diversity and abundance from the ancestral amniote genome [54,55] Certain TEs comprise both autonomous endogenous retroviruses (ERVs), LINE1, Tc-Mariner, or hAT DNA and non-autonomous V-SINE, which are commonly expanded in all amniote genomes [62]. This observation suggests the existence of these TEs in ancestral amniotes. By contrast, several types of TEs have been lost in specific taxa, for example the absence of the Gypsy TE in avian and mammalian genomes [63,64]. Transposable elements are generally scattered throughout the genome [65,66] however, the majority of TEs are abundantly distributed in specific chromosomal regions, such as genomic regions corresponding to G-banding patterns in mammalian species [67], or on sex chromosomes such as LINE1 TEs in mammals, birds, and reptiles [64].

**Figure 2:**
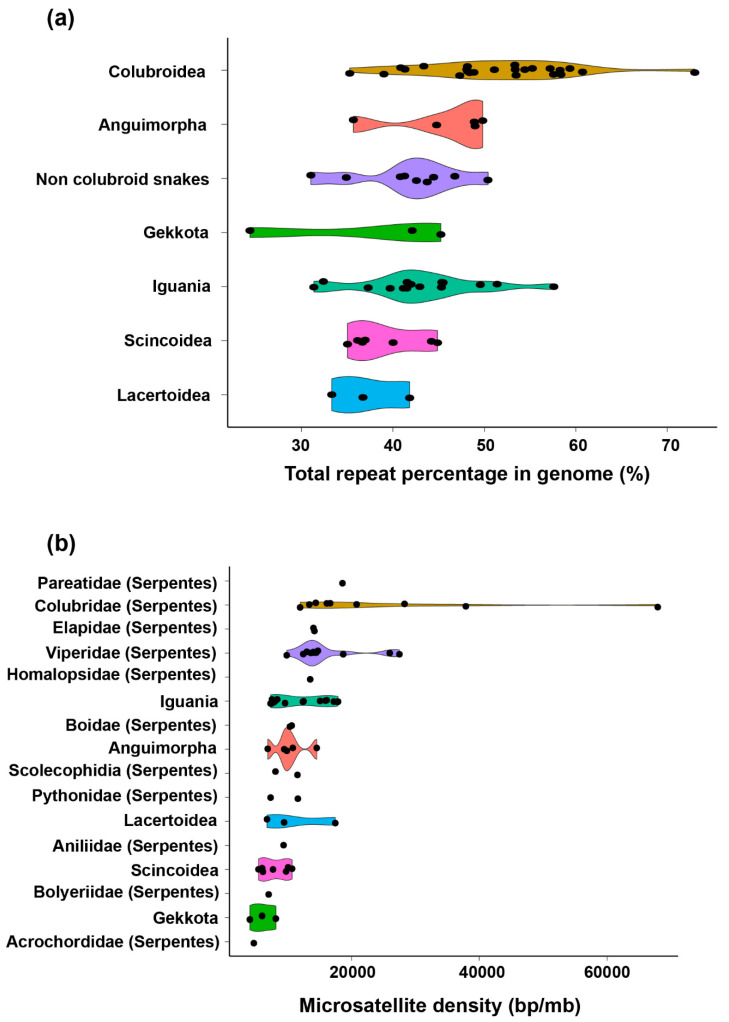
Percentage of total genomic repeats in diverse lineages of squamate reptiles. Violin plots highlighting the family-wise distribution of total repeat proportion (**a**) and microsatellite density (**b**) in genomes. Each dot represents a single species. The species list is given as Supplementary Dataset S1.

Apart from TEs, a high proportion of amniote genomes are satellites that represent large copy number elements arranged tandemly in the heterochromatic region on chromosomes. Satellites can be categorized into different types/families or subfamilies based on the sequence length, structure, organization (including higher-order repeats), and chromosomal localization [36,41,42,44,68,69]. Multiple satellite families are present in a species, but the abundance of families often differs, resulting from the influence of library models with species-specific amplification under selective force [68]. The majority of satellites exhibit a high mutation rate and capability for rapid evolution, and the sequences are highly variable and often clustered as species- or genus-specific satellites as observed in crocodiles, turtles, lacertids, varanids, and snakes [12,29,36,40,69,70,71]. A general assumption about satellite expansion in the genome involves a cohesive evolutionary concept owing to intraspecific homogenization (or concerted evolution) [72,73]. However, satellites with slow mutation rates are present in many snakes and varanids, resulting in a lack of species-specific homogenization [36,70]. Satellite sequences are shared among closely related species, which indicates that the homogenization rate is slower than species divergence mechanisms. The most recent advanced genome-scale investigation of satellites, predominantly microsatellites, has revealed an astonishing abundance in squamate reptile genomes [61,74]. Certain snake species, mainly colubrid snakes, contain higher abundance of overall repeats in their genomes as well as the highest density of microsatellites across all studied squamates [61,75] (Figure 2), in accordance with the amplification of microsatellites on sex chromosomes [20,39,69,76,77,78,79,80,81]. Remarkable variability of microsatellites among the main amniote groups, such as reptiles and mammals, has been previously reported [52,74,82,83] Recent studies have revealed high levels of microsatellite variability within reptile lineages, such as snakes and other squamate reptiles [61] (Figure 2b).

Repeat contents of 28% to 58% are reported for mammalian genomes [84], whereas avian genomes are more compact with repeat contents of approximately 15% [85], which suggests that amniotes show a significant diversity of repeats (Figure 1). Current understanding of repeats and genome evolution of amniotes is biased considerably towards mammals and birds. Mammalian genomes differ from other amniotes in the unique diversity of TEs and abundance of specific elements [64]. In addition, genomes of birds and reptiles show remarkable variety of TEs, most probably derived from the amniote ancestor, whereas limited differences have been observed across major reptilian lineages. Squamate reptiles exhibit a higher degree of repeat variation compared with birds, whereas overall abundance is indicated to be lower than in mammals. Repeat landscapes in squamate reptiles can differ remarkably, even among species within the same genus, with different variation rates, e.g., within the genera *Ophisaurus* (44.8–48.9%), *Coniophanes* (59.4–73%), and *Crotalus* (35.3–47.3%) [36]. The overall repeatomic variation ranges from 24.4% to 73.0% (three-fold variation) in squamate reptiles [61]. It would be interesting to determine repeatomic variation ranges in other amniote groups, such as turtles and crocodiles, and the extent of variation at species and genus levels. Significant advances are possible through large-scale species sequencing and genome assembly.

## 3. Dynamics of TE and Satellite Landscapes in Different Reptilian Lineages

**Figure 3:**
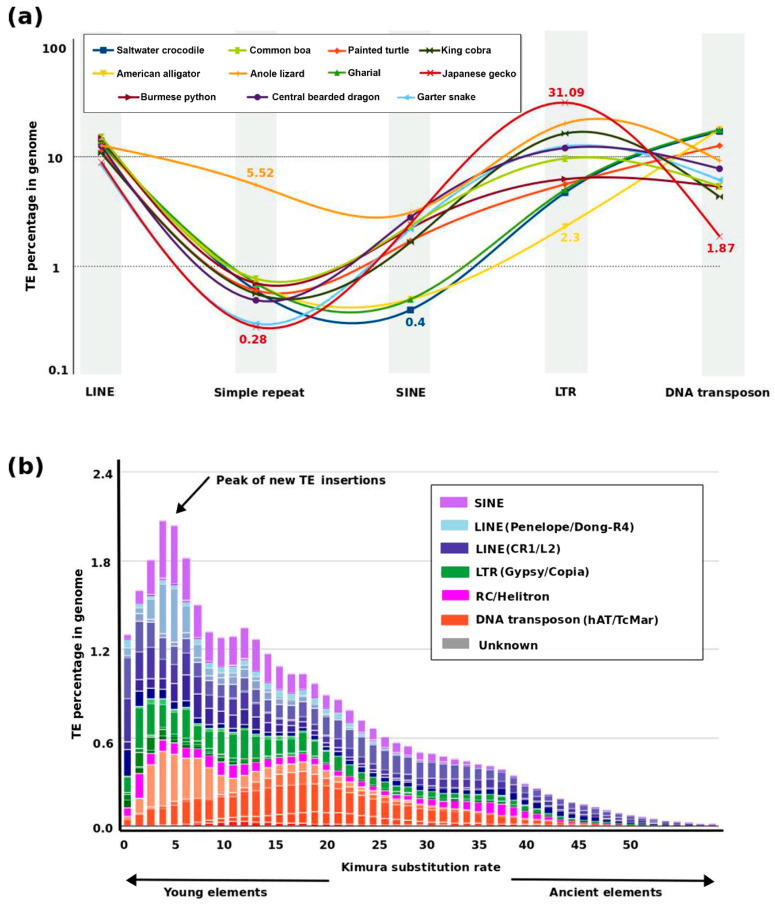
Genomic proportion of repeats in reptiles. (**a**) Comparative line plot of major repeat elements in 11 representative species. The proportion of LINEs is similar for each species, whereas *Anolis* shows the highest abundance of simple repeats. LTRs are most abundant in gecko and least abundant in alligator. Crocodile, gharial, and alligator show similarly low abundance of SINEs. The X-axis has no intrinsic meaning for variable values and is given to represent the types of repeats only. A bar graph of the same data is provided as Supplementary Figure S1. (**b**) Transposable element (TE) evolutionary landscape of the *Anolis* genome. The *y*-axis and *x*-axis represent genomic proportion (%) and Kimura divergence, respectively. A recent wave of transposition in the *Anolis* genome has occurred, as indicated by the black arrow and very low proportions of old elements. K values from 1 to 50 denote evolutionary divergence from younger to older repeats. Data for the percentage of repeat elements was sourced from the literature and the RepeatMasker database (http://www.repeatmasker.org/genomicDatasets/RMGenomicDatasets.html, last accessed, June 2020). The Anole TE landscape was retrieved from RepeatMasker and manually annotated and edited using Inkscape V 0.92 (https://inkscape.org/release/inkscape-0.92/).

Reptile genomes show great considerable TE diversity, with TE family abundance ranging from 23% to 53% within species [49,61] (Figure 1b). The anole lizard mobilome displays extraordinarily diversified TE families annotated as young copies of ancient elements [52,64,86] (Figure 3b). This finding is at odds with avian and mammalian genomes, which show relatively higher enrichment of ancient elements, and suggests that the anole genome underwent an extreme level of recent dispersion of TE insertions [64] (Figure 3). Class I TEs, which are mobilized through retrotransposition mechanisms, represent about 43 families in the anole genome [87,88,89]. In addition, a broad variety of class II TEs do not require a RNA intermediate for movement in the anole genome. These class II TEs are subdivided into several autonomous groups, such as hAT, Mariner, and Helitron, which are indicated to be recent insertions [90]. Three additional TE forms are either extinct (Chapaev) or present in extremely low proportions of the genome (PIF/Harbinger and Polinton/Maverick) [88]. Comparison of TE evolutionary age between snakes and the anole reveals that TEs in snakes are probably older than those of the anole, although earlier expansions of TEs, such as snake1, CR1, LINEs, and BovB, in colubrid snakes were suggested [82,83]. Insertions of TEs, such as hAT-Charlie, Tc1/Mariner, and Gypsy, have also been reported in snake viper genomes, and L2 and CR1 TEs have been detected in boas and pythons [91]. Abundance and diversity of TEs vary considerably among species of archosaurs, especially in birds and crocodiles, and also perhaps in dinosaurs [92,93,94]. Crocodilian genomes possess comparatively higher abundances of TEs than those of birds, which suggests that the former are more similar to archosaur genomes [92,93,95]. Although TE contents vary significantly between crocodilians and birds, CR1s comprise the largest proportion of TEs in both groups [85,92]. CR1 TEs constitute approximately 2–7% of the majority of bird genomes and about 10% of crocodilian genomes [85,92]. Crocodilian genomes also consist of large proportions of other TEs, such as hAT and PIF/Harbinger (7%) and Gypsy (3%) elements [93]. It is hypothesized that the CR1s, ERVs, and SINE activities may contribute to crocodilian diversification [62,96,97]. Apart from crocodiles, understanding the mobilome of turtles has been a focus of research for the past three decades [98]. Recent research has shown that different interspersed elements may share retropositional machinery by exchange of sequence fragments [99]. Although earlier discoveries have shed light on the types of TEs in this monophyletic group, the diversity and level of variation among species remain poorly understood. Recent genomic analysis reported that 10% of the turtle genome may constitute TEs [34,64,100]. Turtle genomes also include CR1/L3 as the most abundant elements of TEs [64,101]. Several CR1 subfamilies have been identified in the turtle genome, exhibiting a lower percentage variation than the consensus sequences, which is indicative of recent expansion of these elements in the turtle lineage [101].

Satellite diversity and abundance are difficult to identify because of repeat complex structures [43,46]. Satellites have been examined in only a small number of reptile species, and knowledge of satellite structure and evolution remains limited. Several studies have focused on chromosome mapping of microsatellites in reptiles [20,45,102], in which the majority of microsatellites were distributed on sex chromosomes. Snakes represent an interesting model to expand our knowledge concerning the evolution of centromeric satellite DNA. Three different types of heterochromatic region-linked satellite families are found in the Burmese python and habu snakes [69]. These satellite families include (1) PFL-MspI (168 bp) from *Protobothrops flavoviridis*, (2) PBI-DdeI (196 bp), and (3) PBI-MspI (174 bp) from *Python bivittatus.* Thongchum and co-workers [36] studied 40 snake species to gain an improved understanding of the conservation of PBI-DdeI satellite evolution and function. Their results indicate high variation in copy number between *P. bivittatus* and other snakes. The PBI-DdeI satellites identified in scaffolds account for approximately 0.353% (5.070 Mb) of the *P. bivittatus* genome, which differs from the copy number estimated by quantitative PCR of approximately 5.73 × 10^6^ copies accounting for 82.53% of the genome [36]. This specific satellite is not identified in any of the genome sequences for snakes, although the PCR approach has successfully detected satellites of many snake species. This suggests that these scaffolds are derived from the centromeric region but are not yet anchored to chromosomes, which reflects the difficulty of sequencing and assembling repeat-rich chromosomal regions. Interestingly, PBI-DdeI satellites are frequently localized to the W sex chromosome of *Naja kaouthia*. Localization of high copy numbers in female rather than male individuals suggests that PBI-DdeI might act as an evolutionary driver with several repeats and facilitate W chromosome differentiation and heterochromatinization [20,21,36]. Satellites have been extensively studied in lacertids [103,104,105,106,107,108,109,110,111], scincids [102,108], and varanids [40,70]. All satellites studied were localized to chromosomal heterochromatin and predominantly in centromeric, pericentromeric, and/or telomeric regions. In Lacertinae, five satellite families, each with a specific phylogenetic distribution, have been identified. Three of these satellite families are genus-specific, i.e., pLHS in *Podarcis* [104], CLsat comprising three subfamilies in *Darevskia* [112,113,114], and Agi160 in *Lacerta* [106,107]. By contrast, the remaining two families are widely distributed in Lacertinae. The satellite pLCS is shared among *Algyroides*, *Teira*, *Lacerta*, and *Podarcis* [103,115], and pGPS is present in *Podarcis*, *Archaeolacerta*, *Algyroides*, *Lacerta*, and *Zootoca* [105]. Giovannotti et al. [108] reported that two satellite families are present in the four species of *Iberolacerta* as (i) the centromeric HindIII family, containing two subfamilies (I and II) representing 5–10% of the genome, and (ii) the TaqI family, possessing only interstitial sites with 2.5–5% of the genome. Differences in abundance, chromosomal position, and evolutionary rate were observed for the HindIII and TaqI families across lacertids. One novel AAN-TaqI satellite with an AT-enriched monomer of 187–199 bp was isolated from populations of Atlas dwarf lizard (*Atlantolacerta andreanskyi*) [110]. This sequence is predominantly localized to the subterminal regions of the short arms of all chromosomes. In *Lacerta*, certain satellites, such as IMO-TaqI, are abundant within heterochromatic regions of the W sex chromosome, which indicates that this repeat may be involved in heterochromatinization and sex chromosome differentiation [109,111]. In varanids, the VSAREP satellite has been identified in water monitor (*Varanus salvator macromaculatus*) and is conserved in the genomes of Asian and Australian varanids but not in African varanids [40,70]. This satellite family is considered to play an important role in chromosomal rearrangement in varanid lineages [70]. In addition, the satellite families CSI-HindIII and CSI-DraI isolated from the Siamese crocodile (*Crocodylus siamensis*) were characterized in the crocodile genome, which indicates their localization in the heterochromatic blocks of centromeres [29]. The CSI-HindIII family is conserved across all extant crocodile lineages of Crocodylidae, Gavialidae, and Alligatoridae. This conservation indicates the possible presence of the CSI-HindIII sequence in the karyotype of a common ancestor of Crocodylia. By contrast, the CSI-DraI satellite is known only in *Crocodylus* and is not represented in other crocodile genomes. This specific occurrence suggests rapid evolution of CSI-DraI and offers insights into how the *Crocodylus* lineage might have diverged from *Tomistoma* and *Gavialis* [8,29,116,117]. In the Chinese soft-shelled turtle (*Pelodiscus sinensis*, Trionychidae), a novel satellite designated PSI-Bgl was cytogenetically characterized and mapped on microchromosomes in the centromere regions and satellite arms but was not detected on macrochromosomes [71]. This site-specific satellite compartmentalization pattern is also observed in the Mexican musk turtle (*Staurotypus triporcatus*) and the giant musk turtle (*S. salvinii*) [118], and suggests that size-specific compartmentalization might have occurred in turtles and also in birds but not in squamate reptiles [24,40,70,119]. Taken together, these recent advances in identification of repeats in reptilian genomes provide a solid foundation for further investigation. To augment knowledge of the dynamics and comparative landmarks of repeats, further in-depth studies are required to understand how the scale of variability of these elements drives genome evolution and how such variation affects processes such as gene regulation, sex chromosome evolution, and karyotype reorganization among reptilian lineages.

## 4. Evolutionary Impact of Repeats in Reptiles: Mediators of Chromosomal Rearrangements to Drive Genome Reorganization

**Figure 4:**
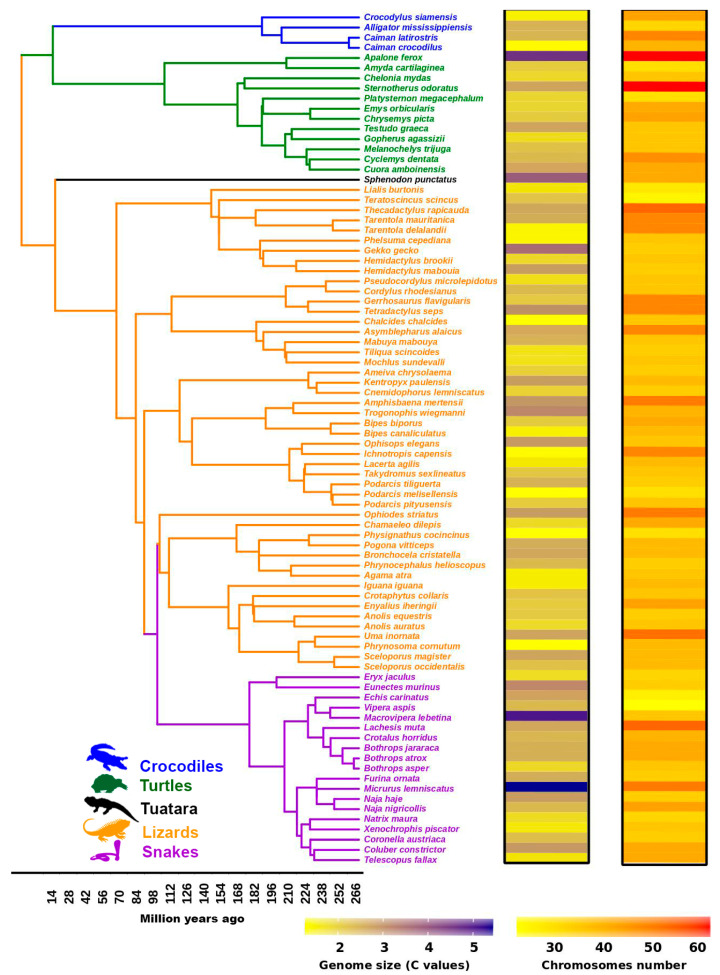
Phylogenetic relationships of 84 reptile species highlighting all families. Chromosome number and genome size were plotted as heatmaps in R using customized script for each corresponding species. The tree topology was retrieved from the TimeTree online database (http://www.timetree.org/). Chromosome number and genome size data were sourced from the animal genome database (https://www.animalgenome.org). Genome size and actual number of chromosomes for each species is listed as Supplementary Dataset S2.

A simple approach is to consider that genome reorganization and chromosomal changes are caused by TE insertions and are mediated through variation in TE copy number throughout the genome and in the species-specific repertoire [84,120]. Such variation is observed in the genome size and structure. Transposable elements are considered to play an important role in the genomic variation among amniotes [64,121,122,123]. These genomic changes are more extensive where these elements can contribute significantly to an increase in genome size, especially in mammals [84]. By contrast, the dynamics of repeats in squamate reptiles challenge the paradigm and existing concept of co-evolution between repeat abundance and genome size [61]. A phylogenetic survey of 84 species comprising five different groups including crocodiles, turtles, tuatara, lizards and snakes showed diverse genome sizes and chromosome numbers (Figure 4), possibly linked with high dynamism of repeats in the reptilian genome. In addition to genome size, TE mobilization and amplification of copy number can affect genome reorganization via non-homologous recombination, leading to diverse types of chromosomal rearrangements, including deletion, inversion, duplication, and translocation, or the emergence of neocentromere and centromere repositioning. This can result in changes in the host genome and diversity at the individual, population, or species level as a consequence of postzygotic reproductive isolation mechanisms [9,124]. In turn, this prevents the formation of fertile offspring through hybrid unviability, sterility, and/or breakdown after fertilization caused by differences in karyotypes and/or chromosome structure between the parental species, resulting in meiotic arrest and subsequent apoptosis of gametocytes [9,125,126]. In a broader context, TE-induced rearrangements contribute to lineage-specific evolution by inducing chromosomal-scale variation, regulation, or mutation of genes, ultimately leading to participation in speciation [127,128,129,130]. A relationship between TE expansion and species divergence has also been hypothesized in mammalian groups, including rodents and bats [51,131,132,133]. Although there is no direct evidence for chromosomal changes mediated by TEs in reptiles [133,134], the incredibly diverse landscape of repeats in reptiles offers potential evidence for prediction of these phenomena under the impact of TE-mediated rearrangements. Multiple independent horizontal transfer (HT) events and peculiar TE patterns may have resulted in extreme genome variation in squamate reptiles [135]. This combination of high-scale transposition and chromosomal rearrangements acted as the major evolutionary force to produce the remarkable species richness and population diversity in this group [14]. The expansion of TEs in a genome may have contributed to the reduced speciation rate in lineages with a large genome size compared with that of lineages with a smaller genome size [136]. Mechanisms of TE accumulation in distinct genomic regions must be understood to explain the role of TEs in evolution. Satellites can also contribute to genome reorganization, such as chromosomal structural changes and heterochromatinization [137]. Centromeric CSI-HindIII identified in the Siamese crocodile is observed on all chromosomes except chromosome 2 [29]. However, linkage homology and the gene order of Siamese crocodile chromosome 2p and the proximal region of 2q are highly conserved with the chicken Z chromosome and squamate reptile chromosome 2p [28]. This might result from centromere repositioning in the Siamese crocodile, leading to the formation of neocentromeres and new centromeric satellites specific to chromosome 2. Although satellites are critically associated with chromosome structural changes, many satellite families exhibit substantial sequence variation among phylogenetically related taxa. Such dynamics can result in acceleration in the rate of mutation and the formation of tandem arrays within a short evolutionary period, leading to speciation [70,138,139]. It is likely that satellites have been continuously linked with fragile sites and/or evolutionary breakpoint regions (EBRs) in various lineages, and associated with frequently occurring chromosomal rearrangements, such as Robertsonian translocation, centric fusion or fission, tandem fusion, and inversion [45,70,140,141,142,143,144,145,146]. Such dynamic behavior of satellites to modulate genomic architecture can be crucial to the promotion of rearrangements. A growing number of studies have provided evidence concerning the role of satellites in reorganization of the genomic architecture and decoding a variety of functions that may link the dynamic nature of these repeats to genome plasticity and evolution [147]. A rapid increase in copy number and divergence of satellites may have contributed to genome evolution through reorganization derived from chromosomal rearrangements [148]. As an example, in reptiles, the genome of rock lizards *Iberolacerta* harbors the HindIII centromeric satellite repeat. This satellite has been linked with chromosomal rearrangements, such as recombination events, which can act as a major evolutionary force in the formation of new repeat monomers, with faster rates of homogenization causing rapid shifts in centromere sequences, triggering species radiation in this lineage [111]. Both TEs and satellites are regarded as crucial actors and as “engines” that trigger genome evolution in reptiles. Genomic regions enriched with such repeats may function as “hotspots” or “fragile-sites” to facilitate rearrangements and drive lineage- or species-specific structural genomic changes that result in phenotypic variation [57,140,143,149,150]. This may also account for the variation responsible for the evolutionary success of amniotes.

## 5. Repeatome and Genome Complexity with Evolutionary Breakpoint Regions

Chromosomal rearrangements are the driving force of chromosome evolution in reptiles, particularly in squamate reptile genomes that exhibit substantial karyotypic variation [14,151]. Crocodile and turtle genomes show lower chromosomal variability and compartmentalization, which might have limited the rearrangement frequency and recombination rate. This lower chromosome variability accounts for the limited change in linkage homology and has maintained the set of genomic elements, and even gene order, although large numbers of microchromosomes are observed in turtles [152,153]. Squamate reptile genomes may thus have acquired smaller chromosomes but retained higher dynamic reorganization [19]. Combination of chromosome painting information with gene mapping and whole-genome data permits us to reconstruct the ancestral karyotype [154,155,156,157,158]. This involves comparison of chromosomes over evolutionary time across amniotes to understand the most likely direction of chromosomal rearrangement in a common ancestor. The tracing of such events can provide insight into evolutionary processes and the role of chromosomal rearrangements in phenotypic evolution and diversity, probably associated with species richness in squamate reptiles [159]. Common types of “gross” chromosomal rearrangement (usually several megabases long) can be detected at the microscope level, such as deletions, inversions, duplications, and translocations (centric fusion and fission, Robertsonian translocation, reciprocal translocation, tandem fusion, terminal transposition, and insertion), although the majority of rearrangements involve multiple fusion [15,16,17,18,19,20,21,28,38,153,160]. Different types of chromosomal rearrangements are involved with structural variation at the scale of the genomic region, ranging from a portion of a single gene to hundreds of genes [161]. Lineage-specific patterns are also observed as centric fusion and fission and tandem fusion, which is common in crocodiles, whereas multiple fusions occur in squamate reptiles [8]. Chromosome morphology can be altered by pericentric inversion that involves breakpoints at different distances on either side of the centromere, or by centromere repositioning, whereby a neocentromere occurs on a chromosome arm without alteration of the gene order [162]. A strong correlation probably exists between EBRs and repeats (TEs, satellites, microsatellites, and multiple gene families) [163,164]. Recently, chromosome-scale assemblies have enabled mechanistic insight into EBRs and intra-chromosomal rearrangements in avian genomes (saker falcon [*Falco cherrug*], budgerigar [*Melopsittacus undulates*], and ostrich [*Struthio camelus*]) [158]. It is necessary to extend these data further for highly rearranged reptilian genomes at such resolutions. While certain chromosomal rearrangements can mostly be induced by repeats through non-homologous recombinations, other rearrangements are associated with unstable genomic regions [165]. Rearrangement polymorphisms in reptiles are correlated with phenotypic differences, which might naturally confer varying fitness in different geographies [166,167].

Such tandem repeats are highly enriched at telomeres and are considered necessary to maintain genomic stability by protecting telomeric regions from degradation [15,19,45,146,168,169]. Apart from their localization at telomeres, these repeats might be embedded within internal sites to form interstitial telomeric sequences, which have been detected in amniote genomes and are considered to be byproducts of ancestral chromosome fusion [170], and are predominantly co-localized with induced chromosome breakage [171,172,173]. Such sequences can trigger genome instability, reshuffling the genomic architecture via different types of chromosomal rearrangements caused mainly by fusion, fission, inversions, or translocations [12,15,20,21,141,146,174]. Interstitial telomeric sequences are considered to be hotspots of chromosome breakage [170] and have been observed in multiple reptile lineages [8,15,20,29,45,77,146,175] associated with chromosomal rearrangements. Repeats might be seeded by transposition or by integration between break-ends from other genomic positions during non-homologous end-joining, an erroneous variant of double-strand break repair. Nucleolus organizer regions (NORs) are highly polymorphic and well known for their potential intragenomic mobility [176]. It is unclear whether unequal recombination or transposition are responsible for this mobility in sex chromosomes of Chinese softshell turtle (*Pelodiscus sinensis*), *S. crassicollis*, *S. triporcatus*, and *S. salvinii* [28,118]. Notably, a common observation is that chromosomal rearrangements occur adjacent to sex-determination loci in different lineages [177], and preferential accumulation of repeats can act to drive the emergence of sex chromosomes, such as Y or W [178]. Why these repeats are preferentially clustered in sex-linked regions remains unclear. One hypothesis states that amplification of these repeats might promote the suppression of recombination and result in the genetic degradation of Y or W and, ultimately, a heteromorphic sex chromosome system might evolve [20,21,36,45,69,177]. Chromosomal changes, including larger inversions and deletions mediated by TE activity, have also been linked to sex chromosome differentiation and evolution [20,179].

## 6. Repeats with Sex Chromosomes in Relation to an Ancestral Amniote Super-Sex Chromosome Evolution Hypothesis

**Figure 5:**
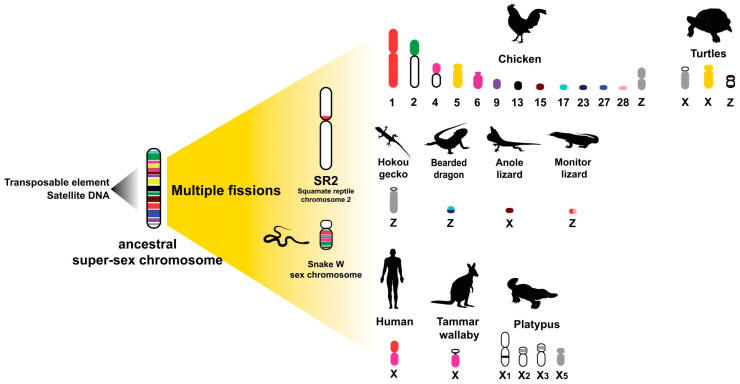
Schematic representation of amniotes sex chromosome evolution. Transposable elements (TEs) mobilization and copy number amplification affected genome reorganization via non-homologous recombination and multiple fission events, resulting in the evolution of heteromorphic X and Y or Z and W chromosomes in different amniote lineages. Chromosomal locations of genes in the amniotes were obtained from comparative gene mapping (chromosome mapping via a cytogenetic technique) and whole genome sequencing as the following sources: chicken (*Gallus gallus*) [24], humans (*Homo sapiens*) and tammar wallaby (*Macropus eugenii*) [195], duck-billed platypus (*Ornithorhynchus anatinus*) [196], green anole (*Anolis carolinensis*) [52], bearded dragon lizard (*Pogona vitticeps*) [191], Hokou gecko (*Gekko hokouensis*) [197], komodo dragon (*Varanus komodoensis*) [198], snakes [20,38], marsh turtle (*Siebenrockiella crassicollis*), wood turtle (*Glyptemys insculpta*), Mexican musk turtle (*Staurotypus triporcatus*), giant musk turtle (*Staurotypus salvinii*), spiny softshell turtle (*Apalone spinifera*), and Chinese softshell turtle (*Pelodiscus sinensis*) [25,26,118,152,199].

Among amniotes, reptiles are ideal for addressing several fundamental biological questions regarding sex determination systems and sex chromosomes, allowing exploration of evolutionary trajectories for sex chromosome differentiation. Sex determination systems in reptiles are diverse and vary among lineages. All crocodiles exhibit temperature sex determination (TSD), and genomes of turtles that exhibit TSD or genetic sex determination (GSD) are now available [34,100]. Combination of short and long read sequencing can provide chromosome-scale descriptions of repeat landscapes of sex chromosomes using all available genome sequence data from turtles as an ancestral archomorph [155]. Such information will provide insight into the origin and degeneration of sex chromosomes, as well as evidence of conservation of repeats on sex chromosomes across taxa. It could also explain the underlying GSD in amniotes, or whether TSD involves structural modifications in DNA adjacent to, or directly concerned with, the sex-determining genomic regions. The ancient tuatara, all crocodilians, a majority of turtles, and some lizards show TSD [8,180,181], whereas most snakes, many lizards, and some turtles exhibit GSD, and a continuum of differentiation between homomorphic and heteromorphic sex chromosomes within taxa is observed [14,182]. Heteromorphic sex chromosomes show accumulation of satellites and amplification of microsatellites or telomeric repeats on Y or W sex chromosomes in many reptilian species and other amniotes [20,45,183,184,185]. In some reptiles the sex chromosome contains no significant enrichment of repeats [185], whereas minimally differentiated XY chromosomes are observed in three cryptodiran turtles (*Staurotypus crassicollis*, *S. triporcatus*, and *S. salvinii*), in which the Y chromosomes are smaller than the X chromosomes owing to a difference in the copy number of 18S–28S rRNA genes [26,118]. One microsatellite amplified on the W chromosome in several caenophidian snakes is the banded krait minor satellite (Bkm), which consists of a microsatellite repeat motif (AGAT)_n_ or (GACA)_n_ sequence, and is associated with the degree of ZW differentiation [186]. Microsatellites on the W chromosome of the banded krait snake (*Bungarus fasciatus*) are also located on the W chromosome of the common tiger snake (*Notechis scutatus*, Elapidae) [185], and are also observed in Kemp’s ridley sea turtle (*Lepidochelys kempii*) and the green turtle (*Chelonia mydas*) as TSD species [187] This results from rapid and independent amplification of repeat sequences on W chromosomes, and suggests that frequent amplification of the repeats has a structural role in heterochromatinization and promotes further sex chromosome differentiation [20]. Similar results have been observed in other amniotes [38], which suggests that amplification of microsatellites has occurred independently in each lineage and might represent convergent sex chromosomal differentiation among amniotes [20,188]. Although sex chromosomes share no homology among amniotes, evidence of linkage homology from several amniotes shows that some overlap of partial sex chromosomal linkage homology is likely to have been part of an ancestral super-sex chromosome [20,21,188,189]. An underlying principle of sex determination in amniote lineages is the sharing of linkage homology, or of sequences such as repeats once linked in a super-sex chromosome that was broken up by different means. Squamate reptile chromosome 2 (SR2) is conserved among squamate reptiles [20,21,186,189], and NORs are generally located on a pair of microchromosomes or chromosome 2 in iguanas and some snakes [175,190]. In addition, NORs are located on the ZW microchromosome in bearded dragon (*Pogona vitticeps*), which shares a common ancestry with SR2 [182,189,191,192]). Two chicken BACs located on *Gallus gallus* chromosome Z (GGAZ), which show high abundance of LINE and LTR TEs, were mapped on SR2 and the snake W chromosome [20,21]. This finding suggests that repeats on the snake W chromosome also share sex chromosomal linkage homology to SR2 and GGAZ. Such repeats (the telomeric sequence, (GATA)_n_, (AAGG)_n_, and (ACAG)_n_) are commonly observed in snake W chromosomes and also in neognathous birds [20,36,38,78,193,194] although the repeats are non-homologous. Similarly, bird and snake W chromosomes share blocks of three repeats (Bkm repeats, 18S-related repeats, and DMRT-related repeats) [185]. These results suggest that repeats are shared partially between the sex chromosomes of chicken and snakes and supports the hypothesis that SR2 and the snake W sex chromosome are associated with a larger ancestral amniote super-sex chromosome (Figure 5) [195,196,197,198,199]. However, the recent chromosome-scale de novo genome assemblies of different vertebrates have not covered this issue, and evidence of chromosome-level genome assemblies is still lacking [200,201]. Although the concept of a super-sex chromosome hypothesis in amniotes has been proposed by a number of cytogenetic based studies evidencing partial linkage homologies, the hypothesis requires improvement for either sex chromosomes of reptiles evolved from a common amniotic ancestral chromosome, or following an independent origin, with a stochastic pattern representing random homologies, where only small sets of genes in a restricted set of species are involved. Convergent evolution of sex chromosomes across distantly related taxa leads to genomic elements, such as repeats, which are particularly adept in a sex-determination role [36]. Are these genes coincidental or are there sequences that serve a selectable function in sex determination in these regions? The hypothesis is not considered as a proven fact and further advanced comparative genomics analysis is recommended. Additional information regarding genomic analysis and transcriptomic activity from squamate reptiles is required to test this hypothesis. Is it possible that amplified microsatellite repeat motifs were retained in the sex chromosomes of a common ancestor, and subsequent reshuffling led to the appearance of sex chromosomes in each lineage?

## 7. Evolutionary Products of Micro- and Macrochromosomal Rearrangements in Reptiles

In addition to the diversity of sex determination systems and sex chromosomes, karyotypes of reptiles constitute a heterogeneous group that is difficult to analyze for chromosome evolution owing to the high degree of variability in chromosome number and composition, even in the absence or presence of microchromosomes [159]. This diversity may help to discover the types and timing of events that contributed to the karyotypes of extant species [200]. On the physical molecular scale, the size of macrochromosomes is generally considered to be greater than 40 Mb and that of microchromosomes less than 20 Mb [196]. It is difficult to trace the evolutionary history of reptile microchromosomes. Microchromosomes were first recorded in iguanid and teiid lizards [202,203,204]. Microchromosomes are considered to have originated from fragments of ancestral macrochromosomes [205]. Different reptiles possess a varying number and presence or absence of microchromosomes in their chromosomal sets. These karyotypic differences are important in reptile comparative analysis for investigating their genetic makeup and variation [206]. Comparative genomic analyses reveal that genetic linkages were highly conserved between avians and reptilians [15,16,17,18,19,20,21,38,52,153,188,189,207,208]. Several crocodile and gecko chromosome pairs are composed of chromosomal segments homologous to turtle and a majority of squamate reptile microchromosomes [8,15,16,20,21,38,153,207]. By contrast, the macro- and microchromosomes of turtles are counterparts of those of chicken, which suggests that the ancestral karyotype of Archosauromorpha, probably composed of at least eight pairs of macrochromosomes and many indistinguishable microchromosomes, has been highly conserved for more than 250 million years following their divergence from Lepidosauromorpha [24,153,209]. A series of chromosomal fusion-fission events (centric fusion-fission, tandem fusions, insertion, and transposition), followed by centromere inactivation events between macrochromosomes or other microchromosomes, resulted in the diversified karyotypes among squamate reptiles [14,15,16,17,18,19,38,45,207]. The phylogenetic placement of reptiles and birds in the presence or absence of microchromosomes suggests that the ancestral karyotype of reptiles might have contained both macro- and microchromosomes [19,52,208]. The microchromosomes disappeared by fusion between macro- and microchromosomes and/or between microchromosomes in the lineage of crocodiles or gecko in squamate reptiles. Chicken and red-eared slider (*Trachemys scripta elegans*, 2*n* = 50) macrochromosomes are remarkably well conserved, considering that these species shared a common ancestor (the Archosauromorpha ancestor) over 200 million years ago [30]. Interestingly, the karyotypic features of the Gila monster, *Heloderma suspectum*, were described by Pokorná et al. [210] consisting of 2*n* = 36 chromosomes (14 macro- and 22 microchromosomes), similar to the Iguania and snake karyotypes [14,211,212]. Microchromosomes might have gained telomeric repeats preferentially. Similar cases are observed in many iguanian lizards and birds [46,213], which suggests that microchromosomes show a higher frequency of recombination than macrochromosomes. In chicken, microchromosomes always show a higher rate of recombination than macrochromosomes [214]. Thus, some regions such as repeats may be functions of the initial copy number and the rate of recombination. Perhaps the rate of recombination might be associated with repeats such as telomeric repeats. However, a genome with a low degree of compartmentalization, which would show limited recombination and a low frequency of chromosomal rearrangements, appears to have been preserved in squamate reptiles, based on the size-specific amplified compartmentalization of satellites, such as microchromosome-specific satellites observed in turtles but not in squamate reptiles [71,166]. Accumulation and conservation of repeats resulted in an increase in chromosome size and number of non-deleterious insertion sites, two features that would have further hampered recombination and chromosomal rearrangements [166]. It would be interesting to determine the crucial impact of chromosomal compartmentalization with species diversity for gecko and lacertids and the remaining groups of squamate reptiles with both macro- and microchromosomes. Crocodylia, which shows low species richness, rarely exhibits genome rearrangements among members, which suggests that the ancestral crocodilian karyotype was highly conserved with no microchromosomes [8,29]. The rate of chromosomal rearrangements may reduce over evolutionary time until genomic stability and an optimal karyotype is achieved. It is hypothesized that both compositional and structural factors of repeats may drive reptilian karyotypic evolution, with transition from the heterozygous to the homozygous phase through a series of rearrangements. For an improved understanding of the underlying mechanisms, characterization of the specific types of rearrangements, such as cryptic inter- or intrachromosomal changes, and comparative genomic analyses in conjunction with cytogenomics or chromosomics are required to investigate genome structure across diverse reptile lineages [215]. Examination of additional reptilian species is needed to elucidate the mechanisms of microchromosome inheritance during evolution.

## 8. Conclusions

The diversity of genomic structural and sequence composition indicates that the reptilian genome harbors an extreme and divergent landscape of repeats compared with other amniotes. Reptiles may, therefore, represent particularly powerful model systems to evaluate hypotheses concerning genomic structure, function, and evolution. Current data show that contents of total repeats in the genome range from 24.37% in *Coleonyx elegans* to 73% in *Coniophanes fissidens*. This variation in the genomic proportion of repeats is much higher than that of other amniotes, particularly birds [61]. We hypothesize that genomic and chromosomal variation of reptiles is correlated with a higher rate of repeat-mediated chromosomal rearrangements. In consideration of how the broader range of genomic repeats impacts on chromosomal dynamics and complexity in reptiles, further studies are encouraged to explain the role of repeats in driving evolution and species radiation. Future research is required to identify any novel lineage-specific repeat families among reptilian taxa and clarify the taxonomic distribution of repeats across species within this group. Do these repeats influence the rate of divergence and could repeat-mediated rearrangements play a detrimental role in causing the extinction of a species? How frequently do chromosomal rearrangements occur in different scales and how do rearrangement rates vary between species and lineages? An integrative approach utilizing molecular phylogenetics, cytogenetics, and modern genomics techniques will assist in determining the extent that repeat elements generate and sustain the remarkable diversity of reptiles. In light of the major contribution of reptile genetics to our understanding of amniote evolution, in-depth insights can be gained by integrative genomics (cytogenomics or chromosomics) to fill existing knowledge gaps from classical cytogenetic approaches [215]. With the availability of NGS technologies and robust bioinformatic tools, we are now in a position to combine modern techniques with classical methods to effectively study chromosome-scale rearrangements at a higher resolution. NGS technology has revolutionized the field of chromosomics. Highly reliable chromosome-level genome assemblies present novel opportunities to decipher previously unresolved evolutionary mechanisms. The integration of modern technologies in future research is strongly recommended to explore the causes and consequences of chromosomal rearrangements and gain mechanistic insights into how these processes have reorganized the reptile genome.

## Supplementary Materials

The following are available online at https://www.mdpi.com/2073-4425/11/7/827/s1. Figure S1. A comparative bar graph of different repeats percentage in the genome of representative reptile species. Supplementary Dataset S1. List of squamates, birds and mammals species with respective percenatge of TEs. Supplementary Dataset S2.
