# Supplementary figures and images for: Consequence of Paradigm Shift with Repeat Landscapes in Reptiles: Powerful Facilitators of Chromosomal Rearrangements for Diversity and Evolution (Running Title: Genomic Impact of Repeats on Chromosomal Dynamics in Reptiles)
Source: Genes (Basel). 2020 Jul 21;11(7):827. doi: 10.3390/genes11070827 (PMC7397244; doi:10.3390/genes11070827)

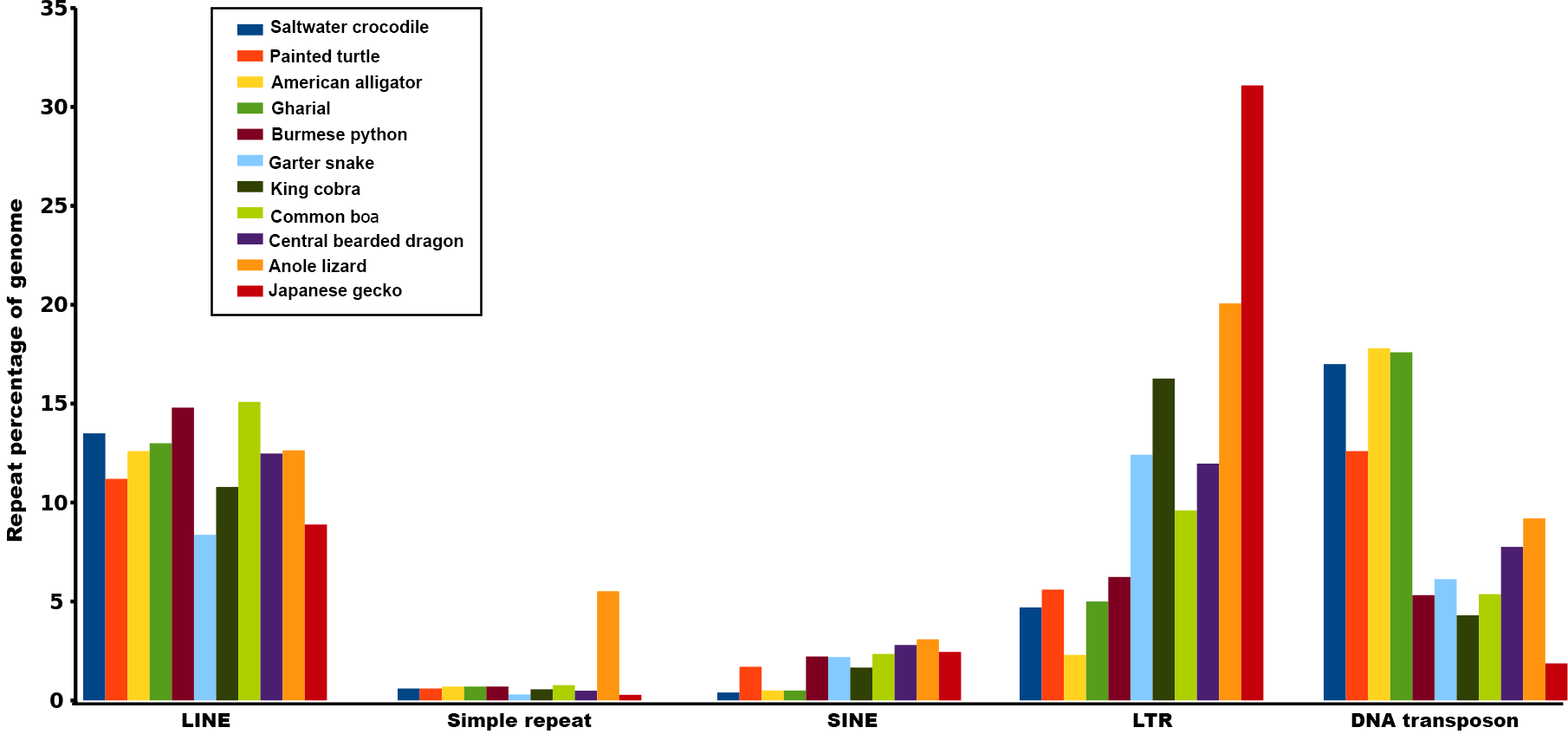

Supplement: Supplementary file 1 [file genes-11-00827-s001.zip › genes-863094-supplementary/Supplementary_figure_1_Round2_Revised.tif]
